# Early exploration of two candidate breast cancer susceptibility genes identified by whole-exome sequencing

**DOI:** 10.1186/1897-4287-10-S2-A91

**Published:** 2012-04-12

**Authors:** R Blanc, A Jammot, T Nguyen-Dumont, ZL Teo, FA Odefrey, F Hammet, DE Goldgar, DJ Park, MC Southey

**Affiliations:** 1 Genetic Epidemiology Laboratory, The University of Melbourne, Melbourne, VIC, Australia; 2 Universite Catholique de Lyon, Lyon, France; 3 Department of Dermatology, University of Utah School of Medicine, Salt Lake City, Utah, USA

## 

Breast cancer (BC) is the most frequently diagnosed cancer in women around the world, and an estimated15-20% of BC cases present with a family history of disease. Genetic variants in known susceptibility genes explain a relatively small proportion of the heritable risk for BC. Genetic variants have been broadly classified into three categories with different levels of risk and prevalence: rare mutations in high-risk genes (e.g. BRCA1, BRCA2); rare mutations in intermediate-risk genes (e.g. CHEK2); and common very modest-risk genetic variants (identified throughout the genome). These categories currently account for 20%, 5% and ~15% of the familial risk, respectively, leaving about 60% of the familial BC risk to be determined.

We have conducted whole-exome capture followed by massive parallel sequencing (XC-MPS)-based analysis on greater than third degree affected relatives from highly selected multiple-case BC families (at least four cases of invasive breast cancer diagnosed before 50 years) which had previously been screened and found not to carry identifiable BRCA1 or BRCA2 mutations.

In this presentation, we focus on two candidate BC susceptibility genes identified by this analysis, each playing a key-role in DNA repair. In both cases, one individual from the pair of cousins was found to carry a predicted protein damaging genetic variant at a consensus splice site. The variants were confirmed and extended analysis within the respective pedigrees performed by Sanger sequencing, subject to DNA sample availability. Further characterization of these variants is being pursued by much larger scale case-control genotyping using the TaqMan platform.

Our work further illustrates the complexities of human genetic variation and the technical and analytical challenges of identifying variation that is associated with inherited predisposition to complex diseases such as breast cancer.

